# Pseudoaneurysm of the Inferior Epigastric Artery Following Laparoscopic Extended Totally Extraperitoneal Repair for Inguinal Hernia

**DOI:** 10.7759/cureus.23377

**Published:** 2022-03-21

**Authors:** Premkumar Balachandran, Senguttuvan Pandian, Swathika V.C., Subbiah T.S., Dakshay A Chordia

**Affiliations:** 1 General Surgery, Apollo Hospitals, Chennai, IND; 2 Interventional Radiology, Apollo Hospitals, Chennai, IND

**Keywords:** etep, laparoscopic inguinal hernia repair, thrombin, inferior epigastric artery, pseudo aneurysm

## Abstract

We hereby report a rare case of pseudoaneurysm of the left Inferior epigastric artery following extended totally extraperitoneal (e-TEP) repair for bilateral inguinal hernia. The patient developed swelling and pain in the lower abdomen one month following surgery. He was diagnosed to have a pseudoaneurysm of the left inferior epigastric artery with significant collection in the retro rectus plane. The pseudoaneurysm was thrombosed using Thrombin injection under ultrasound guidance. He was subsequently taken up for laparoscopic pseudo aneurysm excision with hematoma evacuation and ultrasound-guided transfascial ligation of the inferior epigastric artery with mesh explantation. The pseudoaneurysm was successfully treated and at follow-up, the patient’s symptoms were resolved.

## Introduction

One of the rare complications of surgery of the abdominal wall includes a pseudoaneurysm of the inferior epigastric artery (IEA) with less than 20 cases reported so far [1]. With the more widespread use of minimal access surgery worldwide, more complications are being reported. Abdominal port placement and dissection are the two areas where vessel injury is likely to occur. Due to the rarity of its occurrence and delayed presentation, a high degree of suspicion and timely intervention are required for its treatment [2]. The management of this condition comprises a multi-modality approach which includes conservative management, percutaneous procedures and surgery.

## Case presentation

**Figure 1:**
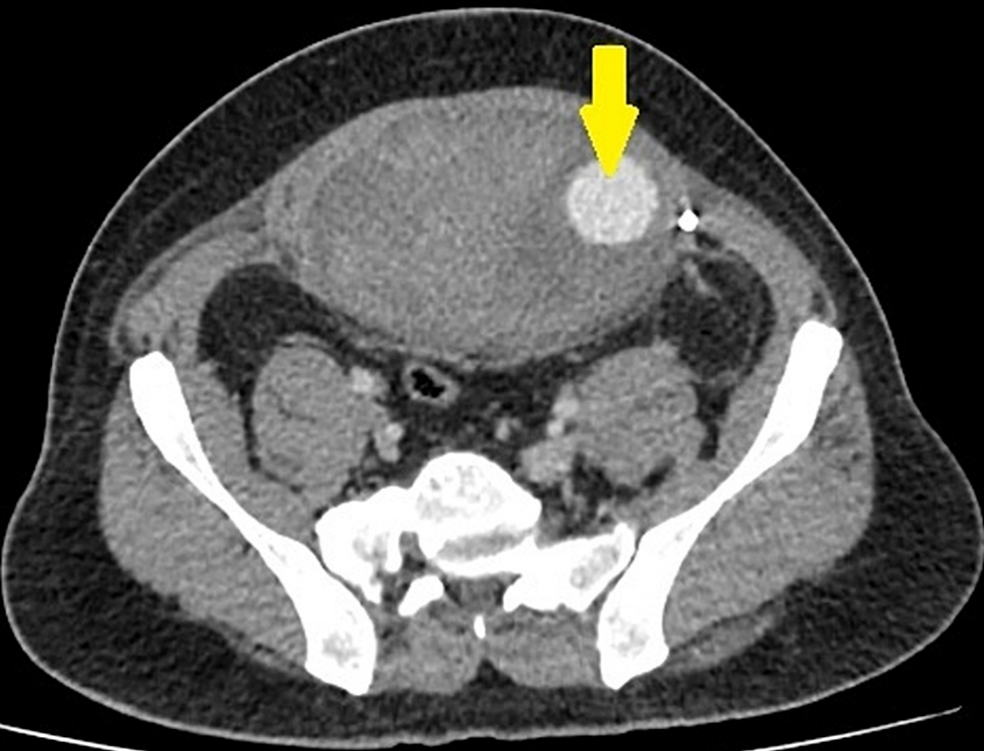
Large retro rectus hematoma of size 8.8 x 11.9 x 14 cm within lower-left rectus sheath extending to the right side across the midline. A 3x3 cm sized pseudoaneurysm arising from the left IEA.

We present a case of a 45-year-old gentleman, with no comorbidities who was admitted with abdominal pain and swelling following Laparoscopic extended totally extraperitoneal inguinal hernia repair done one month ago. The patient was found to have a collection of about 700mL over the mesh on ultrasound and mesh infection was suspected. On further evaluation by contrast-enhanced CT of the abdomen, he was found to have a pseudoaneurysm of size 3x3cm of the left IEA with hematoma of about 700ml anterior to the mesh (Figure 1).

**Figure 2:**
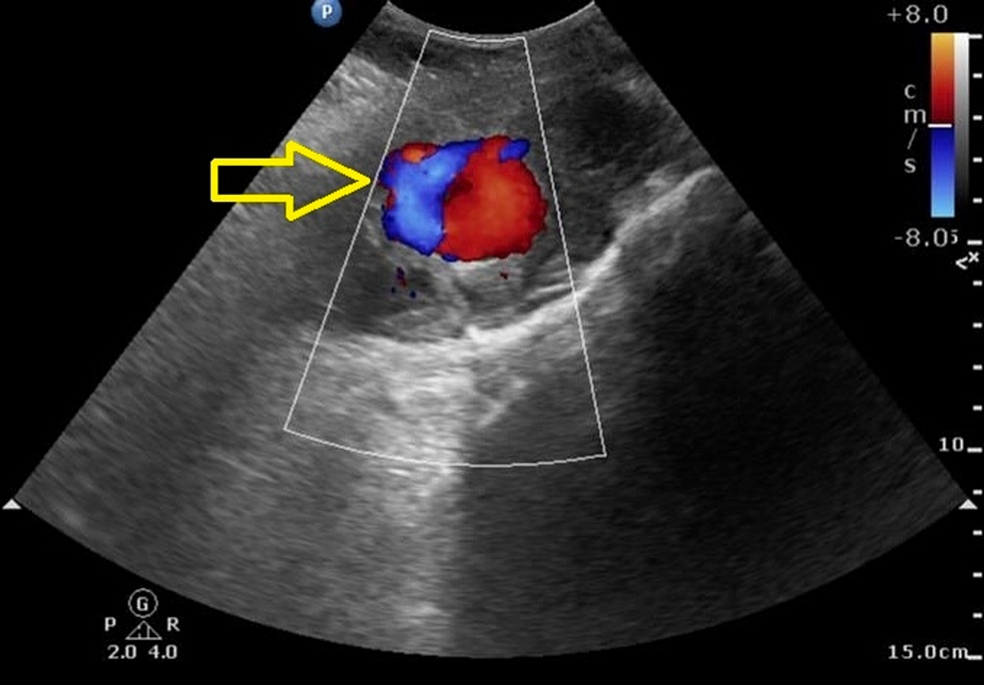
Duplex ultrasound image showing pseudoaneurysm arising from its parent left IEA before thrombin injection.

**Figure 3:**
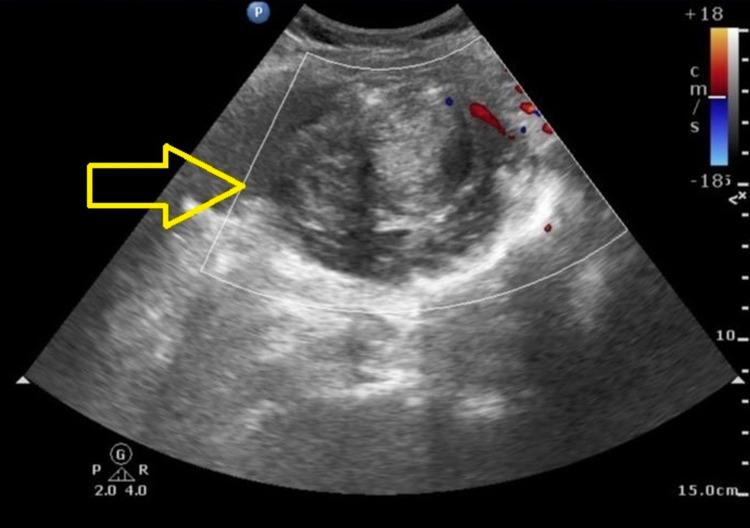
Duplex ultrasound image showing sclerosed pseudoaneurysm of left IEA a minute after thrombin injection.

The pseudo aneurysm was thrombosed using thrombin injection under ultrasound guidance by our interventional radiologist (Figures 2, 3).

**Figure 4:**
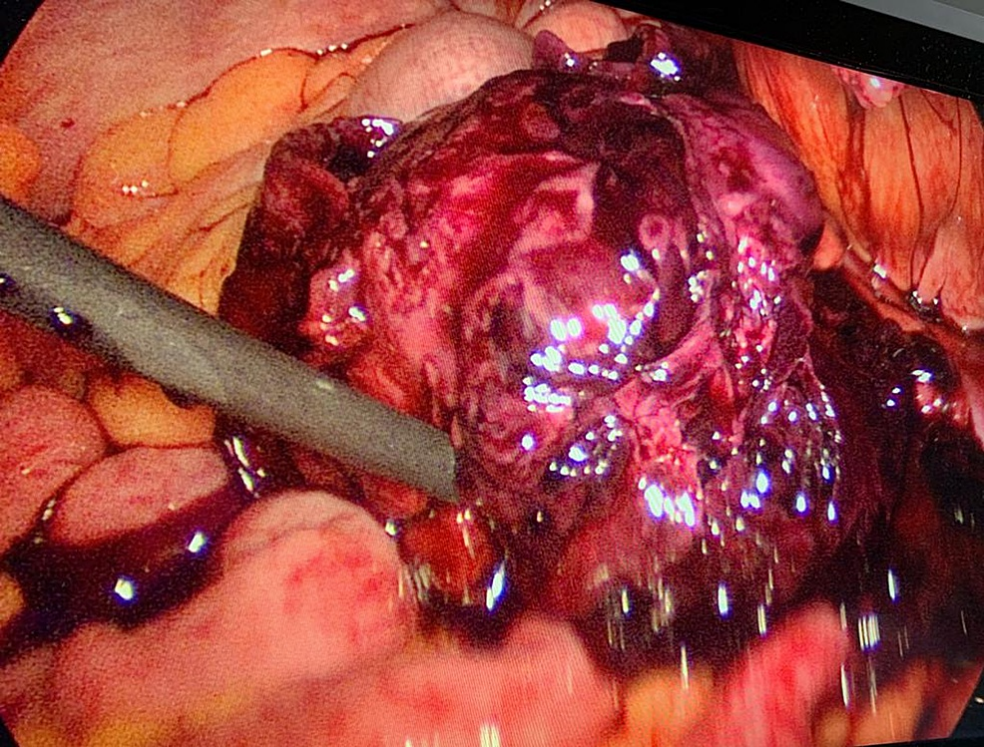
Intraoperative image of the pseudoaneurysm after excision from the left IEA.

Subsequently, two days later, the patient was taken up for a laparoscopic pseudoaneurysm excision with transfascial ligation of the left IEA. Hematoma evacuation and mesh explantation was done as a large hematoma can serve as a nidus for mesh infection. The pseudo aneurysm was very friable and adherent to the hematoma hence it was excised (Figure 4).

**Figure 5:**
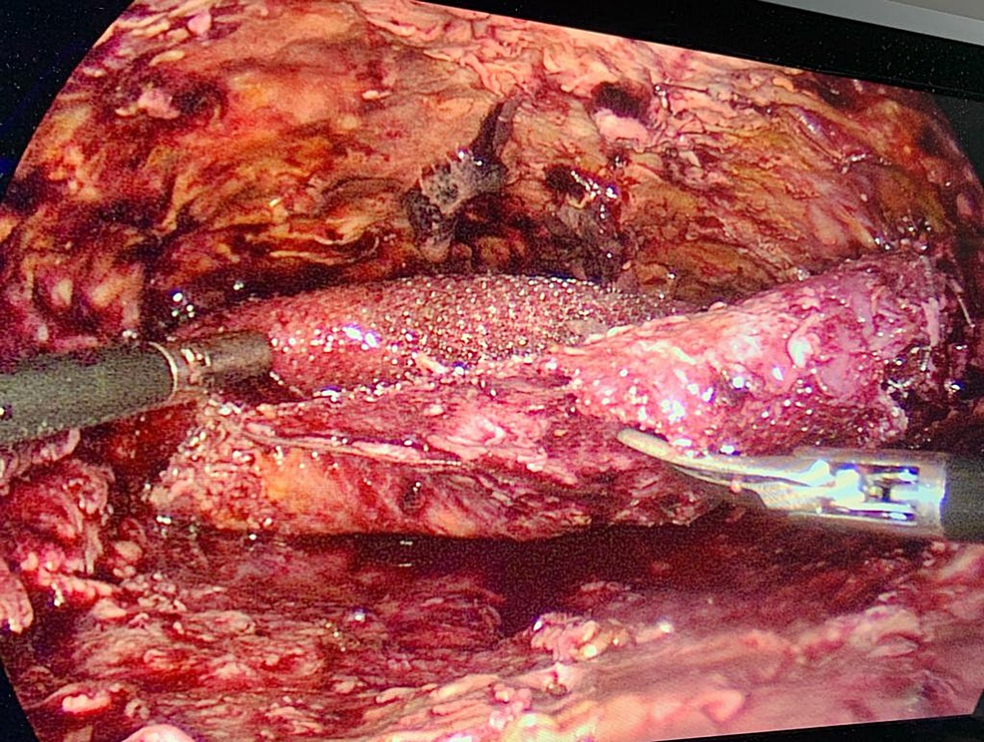
Laparoscopic intraoperative image showing the previous mesh in the retro rectus plane being explanted following excision of pseudoaneurysm.

Following the excision of the pseudoaneurysm, the IEA could not be traced due to altered anatomy from previous surgery. Transfascial ligation of IEA was done under ultrasound guidance. The mesh was removed to reduce the chance of infection as there was some focus of infection around the mesh (Figure 5).

He was discharged with a drain and daily drain output was monitored. The drain was in decreasing trend and was removed on post operative day 5. The patient was counseled regarding the chances of hernia recurrence and the need of surgery if there were any recurrence in the future.

## Discussion

The IEA arises from the external iliac traversing down the posterior wall of the rectus sheath between 4 cm and 8 cm from the midline. The IEA is thus prone to injury on any procedure on the abdominal wall. Incomplete disruption of the vessel wall results in pseudoaneurysms resulting in a partial thickness dilatation due to ligation or trauma. The patient may be asymptomatic or present with a swelling in the lateral abdominal wall with or without pain. On clinical examination, a bruit might be heard over the swelling. They might be detected by color Doppler sonography [3]. In open abdominal procedures, causes of IEA pseudoaneurysm are transfascial suture placement, abdominal wall closure techniques, percutaneous abdominal procedures and blunt injury [2].

In minimally invasive surgery, methods like transillumination, which helps in port placement under direct vision, help in reducing injury to the vessels to 2% [4]. Unfortunately in 18% of cases, inferior epigastric cannot be identified due to obesity and improper vision [4]. Literature enlists laparoscopic surgery and abdominal wound closure as the most common causes of pseudoaneurysms of IEA. There is a wide difference in the presentations and differential diagnoses of hematoma. This makes it quite difficult to diagnose immediately [5]. When detected postoperatively, thrombin injection [6], transcatheter embolization with n-butyl cyanoacrylate [7] have been attempted. But in our case, following thrombin injection, laparoscopic exploration was done in view of the large volume of the collection above the mesh. 

During trocar entry, we must keep in mind that the epigastric vessels traverse the abdominal wall around 4 to 8 cm lateral to the midline and almost parallel to it. Steering clear of this area on either side of the midline will ensure safe entry into the abdomen without injury to the epigastric vessels [8].

## Conclusions

A high degree of suspicion is always required to rule out uncommon complications. Pseudoaneurysm of the IEA is one such complication - rare but having high morbidity. Based on our experience with the current case and existing literature, any lateral swelling following a laparoscopic procedure, a pseudoaneurysm to the IEA must be considered as a differential diagnosis. Early detection by imaging and multimodality timely intervention can result in good outcomes for affected individuals.
